# Adrenal Insufficiency at the Time of COVID-19: A Retrospective Study in Patients Referring to a Tertiary Center

**DOI:** 10.1210/clinem/dgaa793

**Published:** 2020-10-27

**Authors:** Giulia Carosi, Valentina Morelli, Giulia Del Sindaco, Andreea Liliana Serban, Arianna Cremaschi, Sofia Frigerio, Giulia Rodari, Eriselda Profka, Rita Indirli, Roberta Mungari, Veronica Resi, Emanuela Orsi, Emanuele Ferrante, Alessia Dolci, Claudia Giavoli, Maura Arosio, Giovanna Mantovani

**Affiliations:** 1 Endocrinology Unit, Fondazione IRCCS Cà Granda Ospedale Maggiore Policlinico, Milan, Italy; 2 Department of Experimental Medicine, Sapienza University of Rome, Rome, Italy; 3 Department of Clinical Sciences and Community Health, University of Milan, Italy

**Keywords:** adrenal insufficiency, COVID-19, hypopituitarism, infectious diseases

## Abstract

**Context:**

Coronavirus disease 2019 (COVID-19) represents a global health emergency, and infected patients with chronic diseases often present with a severe impairment. Adrenal insufficiency (AI) is supposed to be associated with an increased infection risk, which could trigger an adrenal crisis.

**Objective:**

Our primary aim was to evaluate the incidence of COVID-19 symptoms and complications in AI patients.

**Design and Setting:**

We conducted a retrospective case-control study. All patients were on active follow-up and lived in Lombardy, Italy, one of the most affected areas.

**Patients:**

We enrolled 279 patients with primary and secondary AI and 112 controls (patients with benign pituitary lesions without hormonal alterations). All AI patients had been previously trained to modify their replacement therapy on stress doses.

**Intervention:**

By administering a standardized questionnaire by phone, we collected data on COVID-19 suggestive symptoms and consequences.

**Results:**

In February through April 2020, the prevalence of symptomatic patients (complaining at least 1 symptom of viral infection) was similar between the 2 groups (24% in AI and 22.3% in controls, *P* = 0.79). Highly suggestive COVID-19 symptoms (at least 2 including fever and/or cough) also occurred equally in AI and controls (12.5% in both groups). No patient required hospitalization and no adrenal crisis was reported. Few nasopharyngeal swabs were performed (n = 12), as indicated by sanitary regulations, limiting conclusions on the exact infection rate (2 positive results in AI and none in controls, *P* = 0.52).

**Conclusions:**

AI patients who are adequately treated and trained seem to display the same incidence of COVID-19-suggestive symptoms and disease severity as controls.

Coronavirus disease 2019 (COVID-19) is an infectious respiratory syndrome caused by a novel coronavirus called severe acute respiratory syndrome coronavirus 2 (SARS-CoV-2) (1). It was first identified in Wuhan, China, as responsible for numerous pneumonia cases at the end of 2019. The infection has now spread worldwide, becoming a global health emergency (2). In Italy, Lombardy was the most hard hit region by the pandemic and is still one of the most affected regions in the world, with a prevalence of swab-confirmed infection of 0.75% at the end of April 2020 (3). However, in Lombardy, pharyngeal swab test has been mostly performed in severely symptomatic patients being referred to emergency rooms; thus, the true prevalence of the disease is unknown. The estimated prevalence of infected people based on clinical manifestations in early April 2020 is 19.6% (4).

The clinical presentation of symptomatic infection from SARS-CoV-2 ranges from mild to critical. Most infections are mild; however, 14% and 5% of patients show severe and critical disease, respectively (5-11). In Italy, an overall fatality rate of 13.7% has been registered, ranging from 0.2% in young patients to 31.7% in the elderly (3). The main symptoms reported in affected patients were fever, cough, myalgia or fatigue, and dyspnea; minor symptoms included headache, gastrointestinal symptoms, and conjunctivitis (12).

Risk factors for severe illness are represented by advanced age, smoking, and several comorbidities, such as cardiovascular disease, diabetes mellitus, chronic lung disease, cancer, chronic kidney disease, and obesity (12-17). Immunocompromising conditions and liver disease were proposed as potential risk factors, although specific data are limited (18). Investigations about COVID-19 presentation in specific fragile groups with other chronic diseases have been started (19).

Adrenal insufficiency (AI) is a chronic condition of inadequate cortisol production from different origins (20). As reported by a recent statement released by the European Society of Endocrinology, patients with primary AI (PAI), mainly Addison disease (AD), as well as secondary adrenal insufficiency (SAI), should be considered at risk of being affected by COVID-19 (21, 22). First, because of the overall increased risk of infections reported in AI patients (23), probably as a consequence of their inefficient innate immune response (24), and second, because of the increased mortality rate observed facing respiratory infections, are mainly linked to adrenal crisis exacerbations (25). Some clinical guidelines on how to manage AI patients at the time of COVID-19 pandemic have been issued (22, 26, 27). However, so far, data on COVID-19 presentation and outcome in adrenal insufficient subjects are lacking.

## Materials and Methods

### Study design and participants

We conducted an observational case-control study involving adult patients with chronic adrenal insufficiency, both primary (PAI) and secondary (SAI). As control group, we recruited patients with nonfunctioning pituitary microadenomas (NFPA) and empty sella, without hormonal deficiencies. We made this choice because we expected, in this group, the same incidence of infective diseases as normal subjects and, moreover, most anamnestic data were already present in medical records, avoiding the need for a protracted recruitment phase. Hypoadrenal patients with a previous diagnosis of Cushing syndrome have been excluded from the enrollment considering hypercortisolism a condition highly associated with an increased infection risk that may persist after surgical treatment (28). Patients with 21-hydroxylase deficiency were excluded, too, on the basis of the potential role of hyperandrogenism on the severity of COVID-19 presentation (29-31).

Diagnosis of PAI and SAI was previously performed following international guidelines (32, 33). Other pituitary deficiencies in SAI patients, if present, were adequately replaced with specific hormonal therapies.

Inclusion criteria for both groups were represented by an active follow-up at our endocrinology unit, Ospedale Maggiore Policlinico, in Milan (last visit on site performed in the past 2 years) and by a stable residence in Lombardy region.

All patients’ medical records sorted by name in our center were systematically analyzed, and we extracted all patients presenting the cited inclusion criteria. All patients answered the phone call.

### Methods

From May 1-15, 2020, we retrospectively collected clinical data using phone calls, asking patients to report any symptoms of viral infection that occurred from January 1 to April 30, 2020 and any specific examination performed. According to Italian epidemiological data on COVID-19 outbreak (34), we considered as suggestive for SARS-CoV-2 infection only symptoms that occurred from February 14.

We registered the presence of fever (higher than 37.5°C), cough, myalgia, fatigue, dyspnea, gastrointestinal symptoms, conjunctivitis, anosmia, ageusia, upper respiratory tracts symptoms, or others (thoracic pain, headaches, otalgia), along with the need to increase oral replacement therapy dosage or parenteral administrations of steroids. We collected data on nasopharyngeal swabs and chest imaging results. In the absence of swab and imaging data, we considered highly suggestive (HS) of COVID-19 patients reporting at least 2 symptoms including fever or cough, which are the most frequent. We also analyzed data defining HS symptoms with stricter criteria, including the presence of at least 2 of 3 of the cardinal symptoms: fever, cough, and loss of sense of smell and/or taste. Hospitalization and emergency unit access for suspicious/confirmed COVID-19 infection were also registered.

Data on contacts with infected people, flu shot injection in the winter season, and smoking habits were collected. Working habits acquired from February 2020 were also collected.

Personal data (age and sex), cause of adrenal insufficiency, presence of pituitary deficiencies other than SAI, and the type of replacement therapy were extracted from patients’ medical records.

All AI patients have been contacted at the start of pandemic receiving a brief reminder about keeping adherence to their replacement therapy and adopting sick day rules when indicated. We also invited patients to strictly observe national sanitary rules at work.

The local ethical committee (Fondazione IRCCS Ca’ Granda, Milan) approved the protocol study and patients gave their verbal consent to participate.

### Statistical methods

All categorical data, including diagnosis of COVID-19 and suggestive symptoms, were expressed as percentage (%) and proportion (/) and analyzed using the χ ^2^ test or Fisher exact test when indicated. Continuous parameters with normal distribution were described as mean ± SD and compared using *t* test, whereas non-Gaussian data were expressed using the median and the interquartile range and compared using a nonparametric test (Wilcoxon-Mann-Whitney). Assuming that patients with hypoadrenalism showed a higher incidence of infections than the general population (we considered a relative risk of 2.5) (23), we estimated that at least 102 patients per group needed to be enrolled to detect a significant difference in COVID-19 incidence (with an alpha error of 5% and power of 90%). *P* values < 0.05 were considered as statistically significant. All statistical analyses were performed using SPSS, version 26 (IBM, Cary, NC).

## Results

### Population characteristics

On the basis of inclusion criteria, we enrolled 279 AI patients: 219 SAI and 60 PAI with a mean age 57.5 ± 15.6 and 56.8 ± 15.7, respectively (*P* = 0.754). AI females were 139/279 (49.8%) with a higher prevalence in PAI than SAI (68.3% vs 44.7%, respectively, *P* = 0.001).

The SAI group included 86/219 (39.3%) patients with NFPA, 49/219 (22.4%) with functioning adenomas (29 acromegaly, 16 macro-prolactinomas, and 4 TSH-omas), 47/219 (21.5%) with various pituitary neoplasms (mostly craniopharyngiomas and cystic lesions), 28/219 (12.8%) with other causes of hypopituitarism (mostly idiopathic and posttraumatic), and 9/219 (4.1%) with empty sella. Pituitary hormonal deficiencies, other than SAI, were present in 188/219 (85.8%). Patients with a diagnosis of acromegaly and prolactinoma were well-controlled or in disease remission at the time of investigation.

Among PAI, 47 patients were affected with AD and 13 patients with postsurgical hypoadrenalism; among AD patients, 33 were in the context of an autoimmune polyglandular syndrome type 2.

AI patients were treated with cortisone acetate in 79%, with hydrocortisone (HC) twice or thrice daily in 11.5%, and with modified-release hydrocortisone once daily in 9.5%. A cortisone acetate dose of 25 mg/d was largely used (92.7%), with higher doses in a few patients (7.3%). HC varies from 15 to 25 mg daily (mean daily dose, 20.7 mg) whereas modified-release hydrocortisone dosage varies from 15 to 25 mg daily (mean daily dose, 21.1 mg).

In the control group, 112 patients were recruited (89 patients with NFPA and 23 with empty sella without hormonal alterations as inclusion criteria). Mean age was 57.5 ± 14.3 years and females comprised 52.7% of the sample.

AI and controls’ characteristics (age, sex distribution, smoking, and working habits) were comparable (Table 1). Only the prevalence of flu shots was higher in AI patents, as expected in a fragile group.

**Table 1.** Clinical Features and Collected Data of AI Patients and Controls

	PAI (n = 60)	SAI (n = 219)	*P* ^*1*^	AI (n = 279)	Controls (n = 112)	*P* ^*2*^
Age, y	56.8 ± 15.7 (20-86)	57.5 ± 15.6 (21-94)	0.75	57.3 ± 15.6 (20-94)	57.5 ± 14.3 (27-89)	0.96
Females, n (%)	41 (68.3)	98 (44.7)	0.001^*a*^	139 (49.8)	59 (52.7)	0.65
AI etiology, n (%)						
Addison disease	47 (78.3)		-	-	-	-
Adrenal surgery	13 (21.6)					
Pituitary neoplasms		182 (83.1)				
Other		49 (22.4)				
Replacement therapy						
CO, n (%)	34 (56.7)	187 (85.4)	<0.001^*a*^	221 (79)	-	-
HC, n (%)	13 (21.6)	19 (8.7)	0.01^*a*^	32 (11.5)		
m-HC, n (%)	13 (21.6)	13 (5.9)	0.0007^*a*^	26 (9.5)		
Smoking habit, n (%)	5 (8.3)	38 (17.3)	0.10	43 (15.4)	21 (18.8)	0.45
Flu shot, n (%)	20 (33.3)	79 (36.1)	0.76	99 (35.6)	28 (25)	0.04^*a*^
Symptomatic patients, n (%)	15 (25)	52 (23.7)	0.87	67 (24)	25 (22.3)	0.79
Symptoms duration, d	8.5 (3-19)	7 (3-15)	0.42	5 (3-15)	7 (3-15)	0.65
HS symptoms, n (%)	4 (6.7)	31 (14.2)	0.19	35 (12.5)	14 (12.5)	1.00
HS symptoms duration, d	14 (7-33)	7 (3-15)	0.14	7 (3-15)	15 (7-30)	0.04^*a*^
Active workers, n (%)	10 (16.7)	21 (9.6)	0.05^*a*^	31 (11.1)	13 (11.6)	0.86
High-risk profession, n (%)	5 (8.3)	6 (2.7)	0.06	11 (3.9)	4 (3.6)	0.13
Contacts, n (%)	3 (5)	14 (6.4)	>0.999	17 (6.1)	6 (5.4)	1.00

Age is expressed as mean and range while symptoms duration as median and IQR (interquartile range).

Abbreviations: AI, adrenal insufficiency; CO, cortisone acetate; contacts, direct contacts with infected people; HC, hydrocortisone; HS, highly suggestive symptoms of COVID-19 (at least 2 including fever or cough); m-HC, modified release hydrocortisone; *P*^1^, *P* value PAI vs SAI; *P*^2^, *P* value AI vs controls; PAI, primary adrenal insufficiency; SAI, secondary adrenal insufficiency; symptomatic patients, patients presenting at least 1 suggestive symptom of viral infection.

^*a*^ *P *< 0.05.

### COVID-19 suggestive symptoms and patient management

**Figure 1.**
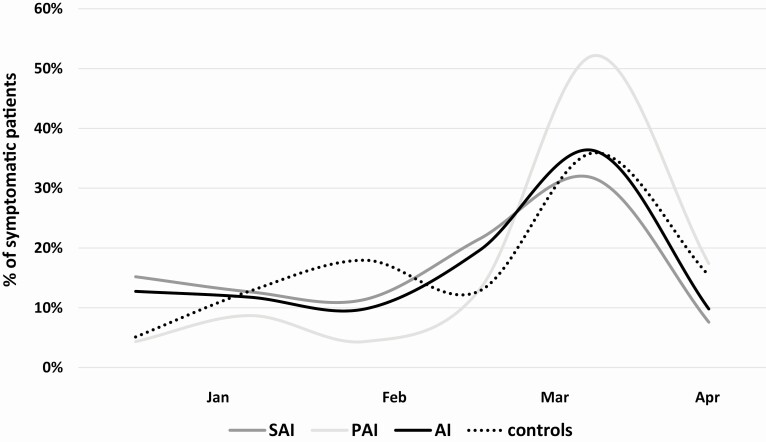
Distribution of symptomatic patients over time, from January to April 2020. We expressed, as a percentage, the ratio between patients who complained symptoms in a specific period and the total of symptomatic patients, in each group. Most patients, both in AI and controls, complained their suggestive symptoms during COVID-19 outbreak, especially on March 2020. AI, patients with adrenal insufficiency; PAI, primary adrenal insufficiency; SAI, secondary adrenal insufficiency.

Symptoms occurrence over time is displayed in Fig. 1. In both AI and control groups, most patients reported the outset in March 2020 (38% and 36%, respectively), whereas another peak of symptoms was reported in controls in the previous February, which overlaps with flu peak period (35) (21% vs 10% of symptomatic controls and AI respectively, *P* = 0.096). Splitting the hypoadrenal group in symptomatic PAI and SAI, we confirmed that most patients in both groups complained about their symptoms during the COVID-19 outbreak (13% vs 21.5% in the second half of February, respectively; *P* = 0.55 and 52% vs 32% in March, respectively, *P* = 0.18).

As stated, only symptoms that occurred from February 14 were considered as suggestive for COVID-19 and thus were included in the following analysis of symptomatic patients.

**Figure 2.**
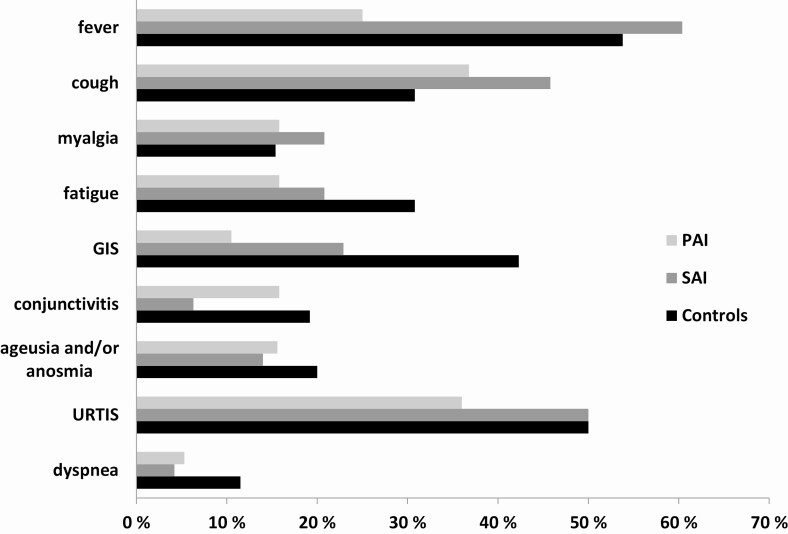
AI, adrenal insufficiency considered as a whole group; GIS, gastrointestinal symptoms; PAI, primary adrenal insufficiency; SAI, secondary adrenal insufficiency; URTIS, upper respiratory tract infection symptoms. *P < 0.05 in controls vs AI; #*P* < 0.05 in SAI vs PAI.

The main clinical features and collected data of both AI patients and controls are shown in Table 1. The prevalence of symptomatic patients (complaining of at least 1 symptom of viral infection, mentioned previously) was similar between the 2 groups (24% in AI and 22.3% in controls, *P* = 0.65). Specific symptoms distribution in the whole AI group, in SAI/PAI, and in controls is represented in Fig. 2. Except for the higher prevalence of gastrointestinal symptoms in controls vs AI patients (42.3% vs 19.4%, respectively; *P* = 0.03), no other differences between these groups were found. Comparing SAI and PAI, only fever prevalence was significantly higher in SAI vs PAI (60.5% vs 26.3% respectively; *P* = 0.02).

The prevalence of HS symptoms was equal in AI and controls (12.5%). Even considering stricter criteria, as defined in the Methods section, the prevalence of HS symptoms were again similar (10.8 % in AI and 8.9 % in controls, *P* > 0.99). A higher prevalence of HS symptoms was observed in SAI than in PAI group, although not statistically significant (14.2% and 6.7%, respectively; *P* = 0.19). Contacts with verified COVID-19 cases was also similar in AI and controls. HS symptom duration was unexpectedly longer in controls than AI (median 15 vs 7, respectively; *P* = 0.04).

In 31.3% symptomatic AI patients, the replacement therapy dose was increased, especially in PAI group (47.4% in PAI and 25% in SAI; *P* = 0.001). The increase was 1.5-fold the regular dose in 42.9% and 2-fold in the others. Only 1 AD patient also practiced intramuscular hydrocortisone injections. Symptomatic patients who did not increase their replacement therapy reported only mild and rapidly improving symptoms. Moreover, most of patients did not consider symptoms as related to COVID-19 at the time of their occurrence. Antibiotics were prescribed by general practitioners in 16.7% symptomatic AI patients and 20.8% controls (*P* = 0.76). No patients required hospitalization and no adrenal crises were reported.

In only 12/279 (4.3%) subjects, a nasopharyngeal swab for SARS-CoV-2 was performed (8 in AI patients and 4 in controls). In 8/12 cases, nasal swab was requested in the presence of HS symptoms and in 4/12 because of contact with COVID-19 patients. Two symptomatic AI patients were found to be positive and none in controls (*P* = 0.52). Chest imaging was requested in 2 symptomatic controls (1.8%) and 5 AI (1.8%), showing a normal result in all patients. Overall, the occurrence of COVID-19 infection was thus confirmed in 0.7% of AI patients and 0% of controls (*P* = 0.52), compared with 0.75% of the general population in Lombardy by the end of April (3).

We did not report any fatal event over the COVID-19 period (from February 14 through April 30). One male patient, 79 years old, with acromegaly and SAI, died in early February (1/279, 0.36%). He was transported to the emergency unit in a comatose condition after several days of gastrointestinal symptoms, started on January 20, 2020. Few data are available on patient management at home on this occasion. Bedside lung ultrasound showed an A pattern excluding B lines, which are typically encountered in COVID-19-infected patients. He displayed atrial fibrillation, and echocardiography showed a severely dilated right atrium with an impaired systolic function. An acute neurologic event was also considered. Based on the time of symptoms occurrence and the clinical presentation, we deemed this event as not related to COVID-19.

## Discussion

AI has been shown to be associated with increased infection risk and immunological alterations (23, 24, 36). These data mainly come from studies involving patients with PAI, whereas no consistent data are available in SAI. It is well known that infections also represent the most frequent trigger for adrenal crisis, which primarily contributes to the increased mortality rate reported in this group (25). Recently, some experts have remarked about the possible risk of COVID-19 contagion and complications in hypoadrenal patients, but the impact of the virus in this group has not been proved. Our specific investigation in this group pointed out some useful information.

According to our data, hypoadrenal patients appeared not to be exposed to a significantly higher risk of manifest COVID-19 typical symptoms than controls even if the exposure risk, based on contacts with infected people and working habits, seemed equal. Also, they did not display severe disease complications that required hospital admission. Along with that, no adrenal crisis was reported. No differences, even in mortality, have been found. Only 1 SAI patient died, but a relationship with SARS-CoV-2 infection was unlikely.

The study group included exclusively patients who were on active follow-up in a dedicated tertiary endocrinological center and thus they have been highly instructed on the possible risks related to infections’ complications and on how to manage their own replacement therapy, if necessary. These characteristics may have contributed to the similar clinical outcome we found in AI and controls.

Epidemiological studies on COVID-19 in Italy reported a peak of notified cases in March 2020, which overlaps with the peak of suggestive symptoms we observed in both the AI group and controls in that month (34). The other peak observed in the previous February, encountered in controls only, might be related to the seasonal flu virus peak (35). The higher number of flu shots in AI patients could explain this difference.

Unexpectedly, we observed a longer duration of HS symptoms in controls vs AI patients along with a higher prevalence of gastrointestinal symptoms. As previously stated, it is possible that AI patients, who are highly instructed on infectious disease complications, adopted a safer behavior, limiting their social contacts and applying better medical managing of virus-related symptoms. Moreover, we could speculate that the high prevalence of gastrointestinal symptoms observed in controls, which is in accordance with the 50% reported in recent studies, may be related to a release of the cytokine in the gastrointestinal tract, a mechanism that could be mitigated by the steroids and by the inefficient innate immune response in AI patients (37).

The main study limitation is represented by the lack of certain diagnostic data on SARS-CoV-2 detection. According to sanitary regulations in Lombardy, nasopharyngeal swabs were performed in specific situations only, namely hospital admissions. The method of data collection, fully based on what patients reported on phone calls, is an additional important study limit. On the other hand, the major strengths are represented by the large number of AI patients recruited, the presence of a control population, and the absence of subjects who did not respond to the call, thus excluding the possibility to have missed hospitalized COVID-positive or dead patients. Future investigations, like serum SARS-CoV-2 antibodies evaluation, will be necessary to clarify the real infection rate in AI patients. A second limitation in our assessment is the incomplete knowledge base at the time of the interview that determined the questions we asked. However, except for cutaneous manifestations (39, 40), all other signs and symptoms resulting today associated with COVID-19 were included (41). Last, we highlight that we decided to consider AI patients as a whole group in our main case-control analyses because, according to our statistical power analyses, the number of patients with PAI was limited.

From our data, we can conclude that at the time of COVID-19 pandemic, patients with AI who are adequately informed on infection-related risks and who are adequately trained on replacement therapy self-adjustment, do not appear to be exposed to an increased risk of developing a more severe clinical impairment than controls.

## Data Availability

Some datasets generated and analyzed during the current study are not publicly available but are available from the corresponding author on reasonable request.

## Abbreviations

AD: Addison disease
AI: adrenal insufficiency
COVID: coronavirus disease 2019
HC: hydrocortisone
HS: highly suggestive
NFPA: nonfunctioning pituitary microadenoma
PAI: primary adrenal insufficiency
SAI: secondary adrenal insufficiency
SARS-CoV-2: severe acute respiratory syndrome coronavirus 2

## Additional Information

***Disclosure Summary:*** The authors declare that no conflicts of interest.
